# Targeting K_v_7 channels in pain pathways

**DOI:** 10.18632/oncotarget.15261

**Published:** 2017-02-10

**Authors:** Ivan Rivera-Arconada, Jorge Vicente-Baz, Jose Antonio Lopez-Garcia

**Affiliations:** Departamento de Biología de Sistemas, Universidad de Alcalá, Alcalá de Henares, Madrid, Spain

**Keywords:** analgesia, M-current activators, ML213, ICA-069673, flupirtine, Neuroscience

The K_v_7 family of potassium channels, formerly known as KCNQ, is composed of five members numbered from 1 to 5. K_v_7 subunits 2 to 5 are expressed in neurons where they give rise to voltage dependent potassium currents (M- or M-like currents or I_M_) with interesting properties, including slow activation kinetics with no inactivation upon depolarization and a complex modulation by an array of neurotransmitters. The I_M_ contributes to setting resting membrane potential and has a strong influence on membrane excitability, acting as a brake that limits firing frequency. The typical I_M_ is associated to heterotetramers of K_v_7.2 and 3 subunits; however other combinations including K_v_7.4 and 5 may add functional and pharmacological diversity. Mutations in four of these subunits are involved in human pathologies such as deafness (K_v_7.4), long QT syndrome (K_v_7.1) and epilepsy (K_v_7.2 and 3), highlighting their great influence in a wide variety of physiological functions. In addition, K_v_7 channels are being extensively studied in relation to anxiety and pathological pain and an interest in K_v_7 regulation of smooth muscle function is growing in later years [1].

Regarding pain processing, K_v_7 channels are expressed in primary afferents as well as in spinal and thalamic neurons. Many studies propose a role for K_v_7 channels in the regulation of nociceptive pathways [2]. The basic tools used routinely in such studies are retigabine, a channel opener, and XE-991, a channel blocker. In neuropathic pain models, retigabine produces a strong attenuation of the abnormal behaviour of axotomized fibres (including spontaneous and stimulus-induced activity) having little effects on normal fibres. This may be due to accumulation of K_v_7.2 subunits in aberrant transduction zones of the neuroma as a consequence of nerve lesion [3]. Unmyelinated nociceptive fibres express K_v_7.5 subunit [4], but no specific targeting of this channel has been tested. Retigabine applied at the spinal cord hyperpolarizes the central endings of afferent fibres. Furthermore, retigabine hyperpolarizes dorsal horn neurons and reduces neuronal excitability. The specific channel conformations responsible for these central effects are unknown to date but there is reason to believe that different conformations may be involved.

K_v_7 openers attenuate nociceptive behaviours in different animal models of pain, including inflammatory, neuropathic and bone cancer pain. Using this latter model some studies show that nociceptive behaviours are reduced after systemic or spinal application of retigabine [5, 6]. It is interesting to note that retigabine effects are completely or partially blocked by XE-991 suggesting the implication of K_v_7 channels. In this line flupirtine, the parent compound of retigabine, has been shown to be more effective than tramadol and pentazocine in double blind clinical trials in patients with cancer pain. All these studies suggest that K_v_7 channels seem especially important in chronic pain. In addition to these observations, two anti-inflammatory compounds commonly used as painkillers, diclofenac and celecoxib, may be active on K_v_7 thus reinforcing the promising role of K_v_7 channels as targets for analgesia.

Retigabine and flupirtine are the only openers of neuronal K_v_7 channels approved for their use in humans, with indications as antiepileptic and analgesic drugs respectively. However, these compounds do not show selectivity for any particular channel configuration and present unspecific effects on other targets like GABA receptors. Given the ubiquity of K_v_7 channels and the diversity of the functions in which they are involved, adverse effects are associated to the clinical use of these basic compounds. However, the therapeutic potential of interacting with these channels has motivated the development of a myriad of compounds and the growth of patents related to their application. In order to develop more potent and selective tools, pharmacological design has followed different strategies including the structural modification of previously characterized compounds and the development of novel structures identified by high-throughput screening [7]. Recently we have tested the ability of two commercially available K_v_7 openers ML213 and ICA-069673 to modulate spinal nociceptive transmission, and compared them with retigabine [8]. ML213 was originally launched as a specific opener for K_v_7.2 and K_v_7.4 subunits and ICA-069673 as a specific opener of K_v_7.2/3 conformations although, unfortunately, other configurations may also be affected according to subsequent reports. In our hands, these compounds seemed to act more specifically on K_v_7 channels than retigabine, since XE-991 was able to completely block their effects even when applied at high concentration. ML213 was the most potent, showing EC_50_ values for spinal reflex depression tenfold lower than the other two compounds. In the other hand, ICA-069673 showed a sort of activity-dependent action that may be explained by an interaction with the voltage-sensing domain of K_v_7 channels. This particular trait may offer an added advantage to the use of this compound in the context of analgesia.

The design of novel compounds with specific pharmacological profiles on different K_v_7 channel conformations provides an exciting opportunity to understand the implication of the channels in a variety of physiological functions including nociceptive transmission. Furthermore, this class of novel compounds is likely to help creating analgesic tools for a variety of chronic pain conditions currently devoid of adequate treatment.
